# Trans-Serosal Multimodal Optical Coherence Tomography for Visualization of Microstructure and Blood Circulation of the Small Intestine Wall

**DOI:** 10.17691/stm2020.12.2.07

**Published:** 2020

**Authors:** M.G. Ryabkov, E.B. Kiseleva, M.S. Baleev, E.L. Bederina, M.A. Sizov, A.N. Vorobyov, A.A. Moiseev, M.M. Karabut, M.A. Plekhanova, N.D. Gladkova

**Affiliations:** Associate Professor, Leading Researcher, University Clinic, Privolzhsky Research Medical University, 10/1 Minin and Pozharsky Square, Nizhny Novgorod, 603005, Russia;; Senior Researcher, Scientific Laboratory of Optical Coherence Tomography, Institute of Experimental Oncology and Biomedical Technologies, Privolzhsky Research Medical University, 10/1 Minin and Pozharsky Square, Nizhny Novgorod, 603005, Russia;; Surgeon, City Clinical Hospital No.30, 85A Berezovskaya St., Nizhny Novgorod, 605157, Russia;; Pathologist, Junior Researcher, University Clinic, Privolzhsky Research Medical University, 10/1 Minin and Pozharsky Square, Nizhny Novgorod, 603005, Russia;; Surgeon, City Clinical Hospital No.30, 85A Berezovskaya St., Nizhny Novgorod, 605157, Russia;; Surgeon, City Clinical Hospital No.30, 85A Berezovskaya St., Nizhny Novgorod, 605157, Russia;; Senior Researcher, Laboratory of Highly Sensitive Optical Measurements, Federal Research Center Institute of Applied Physics of the Russian Academy of Sciences, 46 Ul’yanova St., Nizhny Novgorod, 603950, Russia;; Researcher, Genomics Adaptive Antitumor Immunity Research Laboratory, Privolzhsky Research Medical University, 10/1 Minin and Pozharsky Square, Nizhny Novgorod, 603005, Russia;; Student, Privolzhsky Research Medical University, 10/1 Minin and Pozharsky Square, Nizhny Novgorod, 603005, Russia;; Professor, Head of the Scientific Laboratory of Optical Coherence Tomography, Institute of Experimental Oncology and Biomedical Technologies, Privolzhsky Research Medical University, 10/1 Minin and Pozharsky Square, Nizhny Novgorod, 603005, Russia

**Keywords:** small bowel ischemia, small intestinal strangulation, viability of the small intestine, cross-polarization optical coherence tomography, CP OCT, optical coherence angiography, histomorphometry

## Abstract

**The aim of the study** was to evaluate the performance of trans-serosal multimodal OCT (MM OCT) in *in vivo* detecting of changes in microstructure and blood circulation of the small intestine wall caused by arteriovenous ischemia resulted from intestine strangulation.

**Materials and Methods:**

In experiments on Wistar rats (n=22), we examined the small intestine wall *in vivo* using MM OCT; the access to the intestine was reached through laparotomy. The microvasculature and microstructure of the wall were studied before and after acute arteriovenous ischemia created by ligation of a small bowel segment. The results were then added with data obtained from histological and intravital microscopic examination.

**Results:**

Trans-serous MM OCT allowed us to visualize the bowel wall to its entire thickness, distinguish between the serous-muscular and mucous-submucosal layers, and detect the villi and functioning blood vessels. The structures were best seen after a fat emulsion had been administered into the bowel lumen. In OCT images made in the optical coherent angiography (OCA) mode, large paired vessels (arteries and veins) and micro-vessels with a diameter of >15 μm could be seen. Most of the blood vessels were imaged in the depth range of 80–300 μm from the surface. Capillaries with a diameter of 7–10 μm were not seen, but they produced an overall bright background. In the OCA images reconstructed from a volume of 2.4×2.4×1.8 mm, the total length of the vascular bed before ischemia was 18.3 [16.6; 19.8] mm.

Strangulation of the intestinal loop was associated with changes in the CP OCT picture: the villi-associated vertical pattern and shadows of blood vessels disappeared and the depth of tissue visualization in the cross-channel decreased. The optical equivalents of the serous-muscular layer were preserved; after 180±12 min of ischemia, their proportion in the intestinal wall thickness increased from 25 [18; 32] to 42 [31; 55]% (p=0.031). At that time-point, OCA images of the strangulated bowel loop looked all similar: a uniform dark background with isolated fragmentary large vessels and no signs of blood flow in the microvascular network.

**Conclusion:**

Trans-serous MM OCT provides for *in vivo* visualization of microstructures critical for surgical gastroenterology: the intestinal wall layers including villi and blood vessels of each layer, as confirmed by histological analysis. Destructive processes in the intestinal wall resulting from bowel ligation bring about optical changes, which can be detected using real-time MM OCT.

## Introduction

Successful gastrointestinal (GI) surgery is based on the precise knowledge of the morphological and functional state of the GI tract [1]; recent research in this field provides more evidence on this point [2–5]. The current trends include the development of intravital, minimally invasive label-free techniques in combination with algorithms of data processing and diagnosis making. However, diagnostic options are not equally successful as far as different segments of the GI tract are concerned. One of the reasons for this situation is the objective limitations of modern diagnostic technologies. For example, fluorescence angiography has a limited use because it is unable to detect deeply located vessels [6]. The limited resolution of MRI and positron emission tomography do not allow for the visualization of intramural capillaries. These technologies do not meet the requirements of portability, neither do they provide for multiple repeating of the procedure, which is especially important in surgical gastroenterology [7]. Laser Doppler flowmetry does not include visualization and presents the results in the form of digital data, in which the information of the intestinal wall microstructure is missing [8]. Dark-field microscopy is limited to an imaging depth of 1 mm, which is not enough for viewing the small bowel wall even in laboratory animals [9, 10].

There were reasons to believe that multimodal optical coherence tomography (MM OCT) could become a viable alternative to the traditional methods for studying the intestinal wall. The OCT allows one to obtain real-time images of tissue microstructures smaller than 25 micrometers in a depth of 1.5 mm; OCT run in the angiography mode (OCA) is able to visualize the intramural vascular network and obtain 2D and 3D tissue images [11]. In general, information about tissue structure and blood circulation can be obtained with a resolution close to that of histological biopsy, without the need to take tissue samples or use additional contrast agents [12].

In gastroenterology, OCT has been used mainly for intraluminal endoscopic examination, which made significant progress in studies of the esophagus, stomach, and colon [13–16]. Since the use of endoscopic intraluminal OCT distal to the duodenum and proximal to the colon is technically limited, the small intestine remains the least studied segment of the digestive tract [3]. The shortage of diagnostic capabilities for the small intestine is most noticeable in urgent GI surgery, which requires immediate information about the tissue structure and blood flow before choosing a treatment method.

We hypothesized that MM OCT applied in the transabdominal trans-serous mode, would allow us to obtain objective data on the microstructure and blood circulation in the small intestine wall under normal and ischemic conditions and thus expand the diagnostic arsenal of GI surgery.

**The aim of the study** was to evaluate the performance of trans-serous multimodal OCT in detecting changes in microstructure and blood circulation of the intestinal wall caused by arteriovenous ischemia resulted from small intestine strangulation.

## Materials and Methods

This experimental study was conducted on Wistar rats (males) weighing 210 to 377 g (n=22). When working with animals, we were guided by Order No.199n “On the Approval of the Rules of Good Laboratory Practice” (Russia, 2016) and International Guiding Principles for Biomedical Research Involving Animals (CIOMS and ICLAS, 2012). The ethical principles established by the European Convention for the Protection of Vertebrate Animals used for Experimental and Other Scientific Purposes (Strasbourg, 2006) were followed. Approval for animal experimentation was obtained from the Ethics Committee of the Privolzhsky Research Medical University.

All procedures were performed under general anesthesia induced with a mixture of 3.5% Zoletil and 2% xylazine hydrochloride administered intramuscularly.

In the experiments, we used MM OCT, including the OCA mode and the polarization mode (cross-scattering), to study the intestine wall microvasculature and microstructure before and during arteriovenous ischemia caused by ligation of the intestinal loop. OCT technology is based on the measurement of backscattered low-intensity near-infrared light. In the present study, we used a spectral multimodal optical coherent tomograph (with 1300 nm polarized radiation) developed at the Institute of Applied Physics of the Russian Academy of Sciences (Nizhny Novgorod) [17]. The longitudinal resolution of the system is 10 μm, the resolution depth is 15 μm, the scanning depth in air is up to 2 mm, and the scanning speed — 20,000 A-scans per second. The size of 3D images obtained within 26 s is 2.4×2.4×1.8 mm. The cross-polarization OCT modality (CP OCT) is able to produce two types of images: general tissue structure (both cross-sectional and viewed from above) or images generated only by the components that transform the light polarization to orthogonal. In the OCA mode, variations of the speckle pattern of OCT signal are analyzed; it allows one to visualize blood vessels, including capillaries, with active blood flow [18].

After laparotomy, a fragment of the jejunum located 20–25 cm distal to the duodenojejunal junction was exposed. To minimize the interfering effects of peristaltic movements and respiration-associated intestinal rhythm, the examined loop was placed on a pad fixed near the laparotomy wound. The MM OCT measurements were taken from the side of the serous membrane after fixing the instrument objective on the membrane lateral surface in an area equally distant from the mesenteric and anti-mesenteric edges.

**Figure 1:**
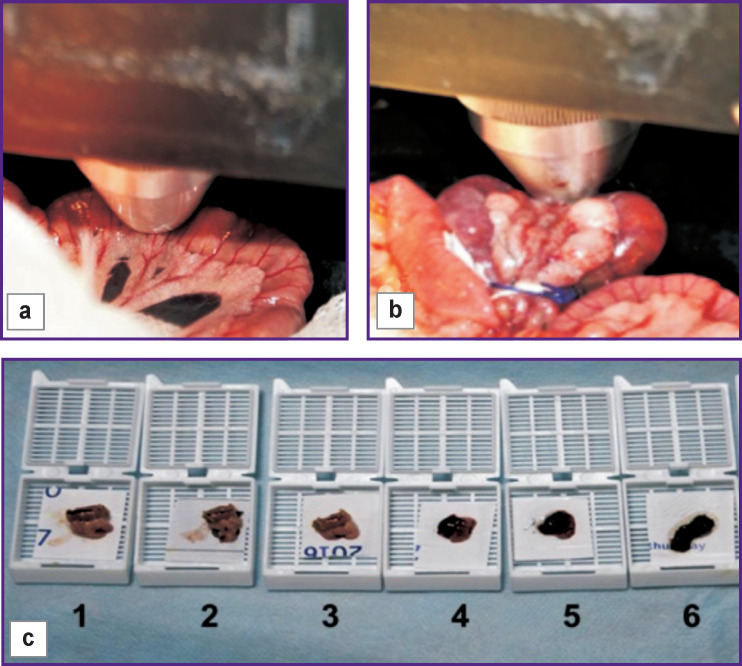
Technical setup for MM OCT investigation of the small intestine wall in rats: (a) position of the MM OCT probe in a normal small intestine; (b) position of the MM OCT probe during intestinal strangulation; (c) samples of the small intestine taken for histological analysis: *1–3* — before strangulation; *4–6* — after 180±12 min of strangulation

In all 22 animals, microstructure and microvasculature of the jejunum segment were evaluated in three segments before any exposure (Figure 1 (a)), then in 2 animals — after the administration of a fat emulsion into the intestinal lumen to contrast the villi, and in 11 animals — after inducing acute arteriovenous ischemia by ligating the jejunum loop and the adjacent mesentery vascular bundle (Figure 1 (b)). We used the term “segment” to denote a section of the intestinal wall of 1 cm long; the distance between two adjacent areas was 1.5–2.0 cm. The length of the ligated section of the intestine was 6.4 [5.5; 6.9] cm. MM OCT scanning was started immediately after the ligation was done; scanning was repeated 180±12 min after that. We recorded two-three 3D images in each zone of the intestine at each time point; a total of 162 images were obtained. To verify the OCA data, images of microvasculature were compared with macrographs of the same area obtained with an Axio Zoom.V16 stereo microscope (Carl Zeiss, Germany).

All sections of the intestine examined by OCT underwent histological verification: 33 samples were taken from 11 animals without any ischemia, 2 of them — after the administration of a fat emulsion; 33 more samples — from 11 animals with intestine ischemia (Figure 1 (c)). Two types of staining were used: hematoxylin/eosin and PAS reaction to assess the integrity of the villous epithelium in the intestinal mucosa (PAS reaction identifies glycogen, neutral glyco- and mucoproteins, sialo-mucoproteins and glycolipids).

We used the ImageJ program to measure the total thickness of the intestinal wall in structural CP OCT images; in addition, we visually assessed the villous mucous membrane. To recalculate the measured thicknesses, we assumed the average refractive index of the intestinal tissue equal to 1.37 [19].

Using OCA images, we calculated the total length of the functioning blood vessels in the intestinal wall without (n=148) and with arteriovenous ischemia (n=140). The methodology for assessing the length of functioning blood vessels has been described earlier [20].

Histological examination (morphometry) was carried out using an Eclipse Ci microscope (DS-Fi2 camera; Nikon, Japan): according to visual criteria, we determined the severity of ischemic and inflammatory lesions, the presence and extent of necrosis for the mucous-submucosal and serous-muscular layers; we also measured the intestinal wall thickness before and after ischemia.

**Statistical data processing** was performed using the Statistica v. 10.0 package (StatSoft Inc., USA) and the Prism 6 software (GraphPad Software, USA). The median values (Me) of the measurements, as well as the upper (Q1) and lower (Q3) quartiles, were calculated. Significance of the differences between the groups was determined using the non-parametric Mann–Whitney test for quantitative parameters. In all cases, the differences were considered statistically significant at a significance level of p<0.05.

## Results

**Figure 2:**
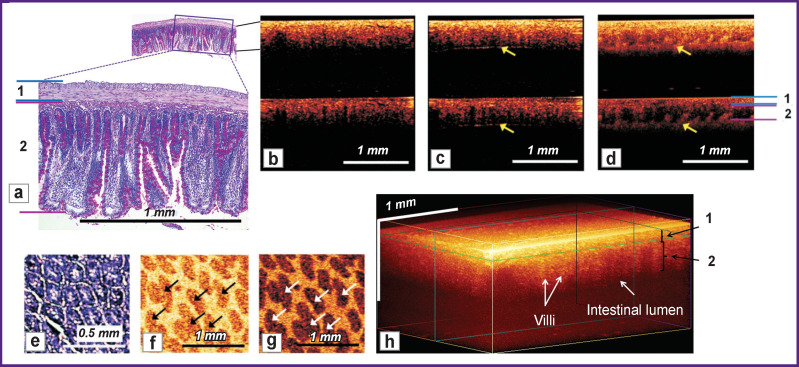
Microstructure of the normal small intestine wall: (a) histological specimen (PAS reaction) corresponding to 2D cross-sectional CP OCT images (b)–(d) obtained from the serous membrane: the upper part is the image in co-polarization, the lower part is made in cross-polarization. *Yellow arrows* indicate the inner border of the intestine containing an air bubble (c) or a fat emulsion (d). *1* — denotes the serous and muscle layers; *2* — submucosal and mucous layers; (e) histological specimen of a cross-section (*en face)* of a villus of the mucous membrane and the corresponding 2D OCT images in co- (f) and cross- (g) polarizations, in which the lower signal villi (*arrows*) are contrasted by a highly scattering medium that fills the spaces between the villi; (h) 3D OCT image

***Microstructure of the small intestine wall at baseline (according to CP OCT data).*** Our study of the small intestine wall using CP OCT resulted in the following structural findings (Figure 2).

1. The intestinal wall is visualized over its entire thickness, from the serous membrane to the lumen (Figure 2 (b)). The administration of air, which creates a sharp boundary of light refraction (Figure 2 (c)), as well as the administration of a fat emulsion into the intestinal lumen, which has higher scattering properties compared to the intestinal villi (Figure 2 (d)), helped establish the exact lower (internal) boundary of the intestinal wall and make sure that the attenuation of the useful OCT signal to the noise level occurs at a depth exceeding the thickness of the intestinal wall. According to CP OCT data, the wall thickness of the rat jejunum is 454 [363; 503] μm, and according to histological data — 855 [790; 915] μm. The difference can be associated, on the one hand, with tissue compression at the time of CP OCT examination, and on the other, with compression of the resected intestinal fragment and simultaneous thickening of all layers of the intestinal wall due to loss of muscle tone.

2. Individual layers (serous, muscular, submucosal and mucous) are not distinguishable in co- or cross-polarization images. This is due, first of all, to the small thicknesses of the outer layers and compression upon their contact with the OCT probe, and also to the similar scattering properties of these tissues. However, the shadows of blood vessels located in the submucosal layer, and the characteristic shape of the villi allow one to visually separate the serous-muscular (Figure 2 (a), “*1*”, see Figure 2 (b), (c)) and mucosal-submucosal layers (Figure 2 (a), “*2*”, see Figure 2 (b), (c)); these layers could be visualized even better, upon injecting the contrast agent that fills the space between the fibers (see Figure 2 (d)). In the cross-polarization image, collagen fibers in the submucosal layer create local spots of high cross-scattering and thus help to identify this layer. According to the CP OCT data, the submucosal layer and the mucous membrane together comprise 75 [68; 82]% of the total thickness of the intestinal wall; according to the histological data, this fraction is 86 [82; 88]%.

3. The quality of visualization of the mucosal villi varies significantly with conditions of the OCT scan and the method of presenting OCT data. In the cross-sectional 2D CP OCT images, the villi are clearly visualized only when a fat emulsion (contrast agent) is administered into the intestinal lumen (see Figure 2 (d)). In the 2D image *en face* (Figure 2 (f), (g)), the transverse section of the villi looks strongly similar to the histological picture of the intestinal wall (Figure 2 (e)). In the 3D OCT image of the intestinal wall, the villi look vertically stratified, but the remaining layers have no clear boundaries (Figure 2 (h)).

**Figure 3:**
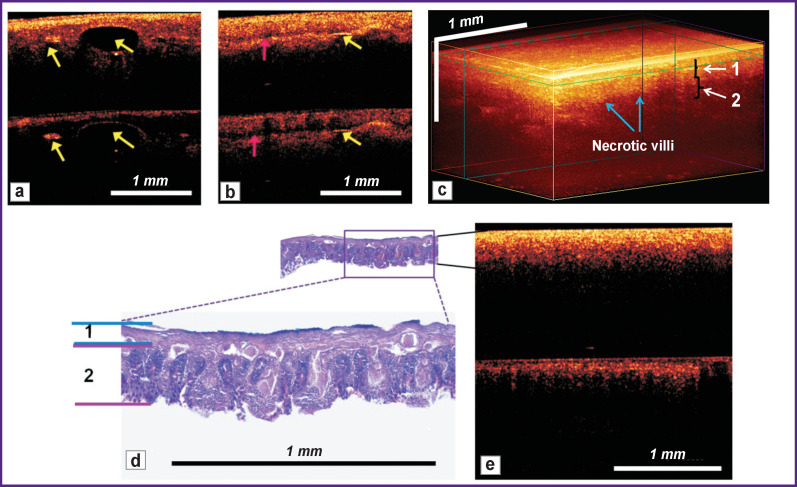
Microstructure of the small intestine wall after strangulation: (a), (b), (e) 2D cross-sectional CP OCT images obtained from the serous membrane of the strangulated intestine: the upper part is the image in co-polarization, and the lower part — in cross-polarization. *Yellow arrows* indicate gas bubbles in the intestinal lumen; *pink arrows* — the narrowed intestinal lumen, exposing the opposite part of the wall; (c) 3D OCT image; (d) histological preparation (PAS reaction) corresponding to this image (e). *1 —* indicates the serous and muscle layers; *2* — submucosal and mucous layers

***Microstructure of the small intestine wall during prolonged strangulation (according to CP OCT data)*.** Strangulation of the intestine loop led to dramatic changes in CP OCT images. The characteristic vertical pattern of the villous area disappeared because of progressive necrosis and the developing absence of shadows from blood vessels; these changes manifested in decreasing depth of tissue visualization via the cross-channel (Figure 3 (a), (b), (e)). In addition, gas bubbles appeared in the intestinal lumen (see Figure 3 (a), (b)); over time, the opposite side of the intestinal wall became visible (see Figure 3 (b)). After 180 min of strangulation, the optical equivalents of the serous membrane and muscle layer were preserved, and their fraction in the total thickness of the intestinal wall increased from 25 [18; 32] to 42 [31; 55]% (p=0.031); the proportion of submucosal and mucous membranes, on the contrary, decreased from 75 [68; 82] to 58 [45; 69]% (p=0.028). In 3D images of strangulated intestine, the optical equivalents of an individual villus (unlike the normal intestinal wall) could not be visualized (Figure 3 (c)).

Similar to the CP OCT images, in histological specimens, the total intestinal wall thickness of the strangulated loop (601 [509; 786] μm) was less than that in the intact intestine. According to a selective analysis, there was an increase in the serous and muscle layers, which were thicker than normal by 2.4 times (p=0.001) due to edema and severe venous hyperemia of the tissues. There was also a 1.6-fold increase in the ratio of serous-muscular to mucous-submucosal layer thickness, mainly due to the reduced thickness of the mucosa caused by necrosis and destruction of the villi (Figure 3 (d)).

**Figure 4:**
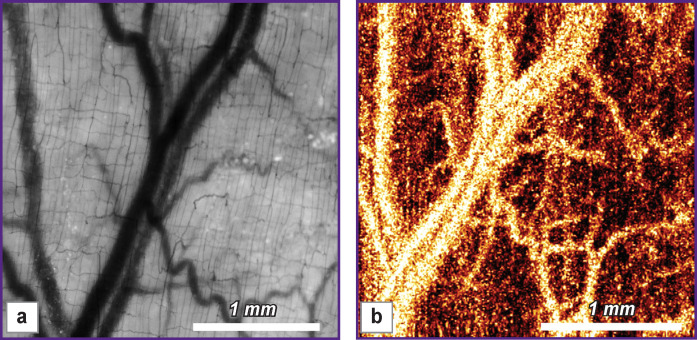
Comparative images of the microcirculatory bed of the normal small intestine, obtained by light microscopy (a) and OCA (b) of the serous membrane There is a consistent pattern (presence and relative position) of large and smaller blood vessels in both images

**Figure 5:**
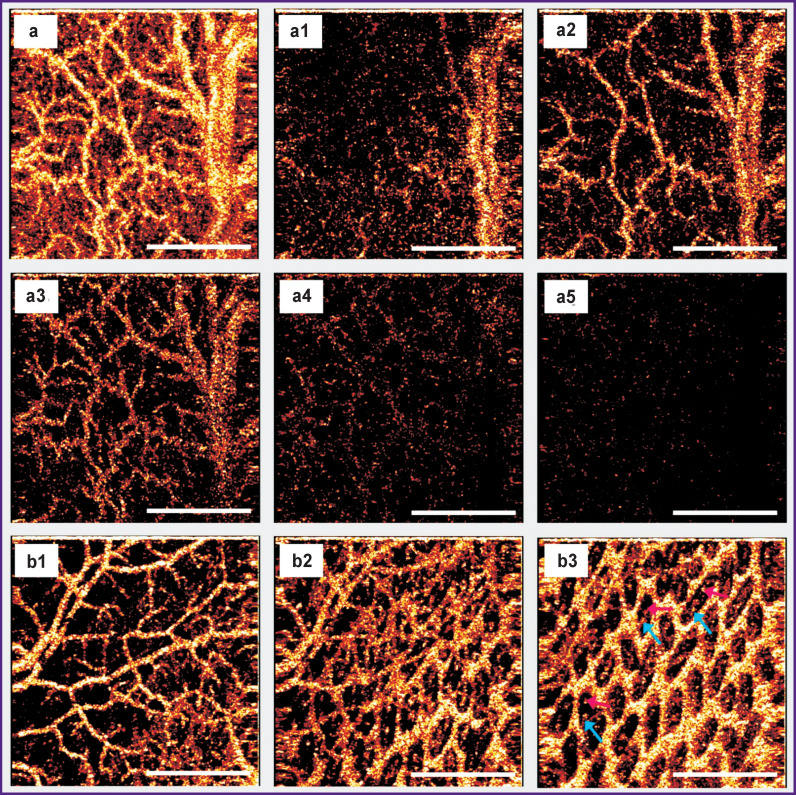
The intramural vascular network of the normal rat small intestine visualized by the OCA method: (a) a network of blood vessels built over the entire volume of OCT data (z=1800 μm), (a1)–(a5) sequential OCA images constructed over part of the OCT volume (z=150 μm) from the sub-serous layer to the mucous membrane at a depth of 10–160 μm (a1), 80–230 μm (a2), 150–300 μm (a3), 220–370 μm (a4), 290–440 μm (a5). The bulk of blood vessels is visualized in the depth range of 80–300 μm from the surface (images (a2), (a3)), which corresponds to the lower part of the muscle layer, submucosal and mucous layers, including the base of the villi; (b1)–(b3) sequential OCA images constructed over part of the OCT volume (z=150 μm) at the time-point when the fat emulsion was inside the intestinal lumen: at the level of the serous, muscle and part of the submucosal layers (b1); at the level of the submucosal and mucous layers (b2), where bases of the villi are contrasted against the bright background in the lower right corner; at the level of the villi of the mucous membrane (b3), where one can see arterioles and venules located inside the villi (bright points are indicated by *blue* and *pink arrows*). Bar — 1 mm

***Blood circulation in the normal small intestine wall (according to OCA).*** In OCA images of the normal small intestine, numerous blood vessels of various diameters were visualized; among them, large paired vessels (with a larger diameter — a vein, with a smaller one — an artery) and a microvascular network evenly distributed over the image (Figure 4 (b), Figure 5 (a)). Verification of blood vessels in such images was carried out by comparison with digital microscopic photographs obtained from the same segment of the intestinal wall. The presence and relative position of large and smaller blood vessels matched each other in these two methods (Figure 4). However, in comparison with microscopic images clearly showing the capillary network, the OCA images could not identify capillaries in their true diameter of 7–10 μm; instead (due to the resolution of 15 μm only), these small capillaries created an overall bright background. A quantitative analysis of OCA images showed that the total length of the vascular bed reconstructed from a volume of 2.4×2.4×1.8 mm was normally 18.3 [16.6; 19.8] mm.

**Figure 6:**
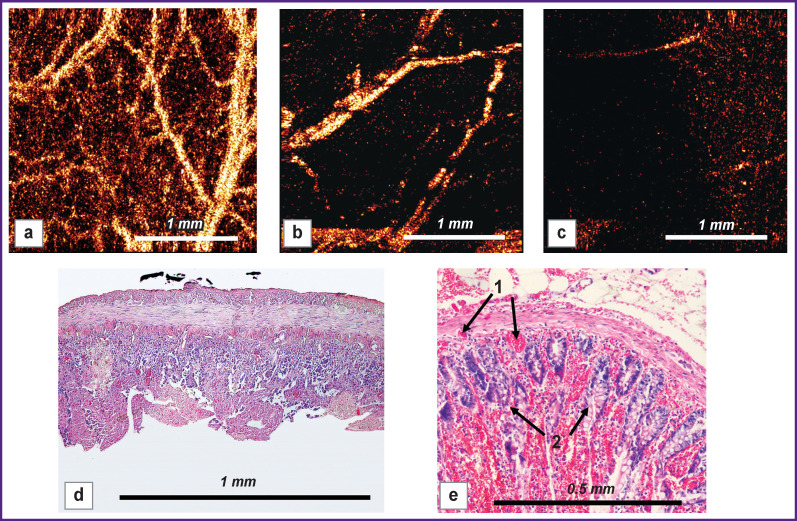
**The intramural vascular network of the small intestine under prolonged strangulation:** (a)–(c): OCA images made before strangulation (a), immediately after ligation (b) and 180 min after it (c); after strangulation of the intestinal wall, a noticeable decrease in the number of visualized blood vessels is observed (b); over time, almost all blood vessels disappear from the OCA images (c); (d), (e): respective (c) histological preparations at low (d) and high (e) magnifications, where necrosis of most villi of the mucous membrane (*1*) and part of the submucosal layer is observed; stasis and blood clots (*2*) in blood vessels of the mucosa and submucosal membranes; hematoxylin/eosin staining

By changing the volume of OCT data in depth (by setting different z values) from which the network of blood vessels is built, it is possible to visualize vessels at different depths (Figure 5 (a1)–(a5), Figure 6). According to OCA images (a1)–(a5) in Figure 5, the bulk of blood vessels is visualized in the depth range of 80–300 μm from the tissue surface (see Figure 5 (a2), (a3)); this depth corresponds to the lower part of the muscle layer, submucosal and mucous layers, including the base of the villi. In addition, with a contrasting fat emulsion it was possible to visualize capillaries of the villi in cross-section images (*en face*) (Figure 5 (b1)–(b3)).

***Microcirculation of the small intestine wall during its prolonged strangulation (according to OCA)*.** Immediately after the intestine loop strangulation, the OCA images showed a noticeable decrease in the number of visualized blood vessels; the background blackened due to the cessation of blood circulation and red cell stasis transforming into blood clots. After 180 min, all OCA images of the strangulated loop showed the same picture: a uniform dark background with isolated fragmented large arteries; there were no signs of blood flow in the major part of the microvascular network. In histological specimens of the strangulated intestine, signs of pronounced venous congestion were found: there were markedly expanded thrombosed veins in the submucosal and serous-muscular layers of the intestinal wall (Figure 6 (d), (e)).

The total length of the functioning vessels 180 min after the strangulation was 89.2±4.7% lower than the baseline (p=0.001) and amounted to 1.7 [0.8; 3.5] mm.

Thus, the present results confirm the possibility of identifying functioning arteries and veins located at various depths: from relatively large sub-serous vessels to arterioles and venules of the mucous membrane villi.

## Discussion

The search for optimal diagnostic methods capable of assessing the structure and function of the small intestine wall is being continued. None of the traditional methods fully meets the requirements for intraoperative diagnosis: non-invasiveness, label-free measurement, high resolution (at the level of cells or groups of cells), the possibility of multiple repetition, preservation and digital processing of the data obtained [21]. The complexity of the task is compounded by the fact that the structure and functional activity of the circulatory network of the small intestine wall can quickly change over time under acute diseases and injuries [5, 10, 22]. The present *in vivo* data of the CP OCT method concerning the damage to vasculature and structure of the intestinal wall during arteriovenous ischemia are consistent with the results of a recent study [23] where fluorescence microscopy, laser Doppler flowmetry, and histological analysis were used.

Since the first p1ublications on using OCT (early 1990s), endoscopic devices for OCT measurements have been steadily improved [24, 25]. In gastroenterology, OCT endoscopic instruments are used primarily for probing the microstructure and blood circulation in the GI walls: the esophagus, stomach, biliary tract, and some segments of the intestine [3, 15, 16]. However, compared with the rest of the GI tract, the small intestine remains the most “problematic” and least studied digestive organ. Previously reported endoscopic OCT studies provided information about intestinal wall only in the proximal and distal parts of the small intestine — the duodenum and terminal ileum [26–29]. Other options of intraluminal endo-OCT related to the small intestine are limited by the current possibilities of endoscopic technique: for modern duodeno- and the terminal ileum are accessible. Balloon enteroscopy is not widely used in emergency bowel surgery; capsular intestinal microscopy has not yet been technically combined with OCT.

Trans-serous OCT can potentially provide surgeons and scientists with data on the condition of the intestinal wall intraoperatively, throughout the entire area of interest, without the need for an endoscope to be inserted into the organ lumen. The first data obtained in this study of the small intestine wall need to be systematized and expanded in order to become a reliable basis for diagnosing diseases of the small intestine.

## Conclusion

Trans-serous multimodal OCT allows *in vivo* visualization of microstructures critical for decision-making in surgical gastroenterology; these structures include all layers of the intestinal wall, villi of the mucous membrane, and functioning blood vessels of all layers of the intestine.

Acute changes in the intestine wall, resulted from its strangulation, cause changes in the optical properties of the tissue and can be diagnosed using multimodal real-time OCT.
